# Evaluation of Anti-HPV18 Antibody Titers Preceding an Incident Cervical HPV18/45 Infection

**DOI:** 10.3390/vaccines13070722

**Published:** 2025-07-02

**Authors:** Fanua Wiek, Viswanathan Shankar, Ana Gradissimo, Angela Diaz, Ligia A. Pinto, Nicolas F. Schlecht, Robert D. Burk

**Affiliations:** 1 Department of Pediatrics, Albert Einstein College of Medicine, 1300 Morris Park Avenue, Forchheimer Building, Room 109, Bronx, NY 10461, USA; flora.wiek@einsteinmed.edu; 2 Department of Epidemiology & Population Health, Albert Einstein College of Medicine, 1300 Morris Park Avenue, Block Building, Room 315, Bronx, NY 10461, USA; shankar.viswanathan@baystatehealth.org (V.S.); nicolas.schlecht@roswellpark.org (N.F.S.); 3 Department of Healthcare Delivery and Population Sciences, University of Massachusetts Chan Medical School-Baystate, 3601 Main Street, Springfield, MA 01107, USA; 4 Sloan Kettering Institute, Memorial Sloan Kettering Cancer Center, New York, NY 10065, USA; gradisa@mskcc.org; 5 Department of Pediatrics, Icahn School of Medicine, Mount Sinai Adolescent Health Center, 312-320 East 94th St., New York, NY 10028, USA; angela.diaz@mountsinai.org; 6 HPV Serology Laboratory, Frederick National Laboratory for Cancer Research, Frederick, MD 21701, USA; pintol@mail.nih.gov; 7 Department of Cancer Prevention and Control, Roswell Park Comprehensive Cancer Center, Elm & Carlton Streets, Buffalo, NY 14263, USA; 8 Departments of Microbiology & Immunology, and Obstetrics, Gynecology & Women’s Health, Albert Einstein College of Medicine, 1300 Morris Park Avenue, Forchheimer Building, Room 109, Bronx, NY 10461, USA

**Keywords:** HPV18, papillomavirus, quadrivalent vaccine, antibody titer, VLP

## Abstract

**Background:** The Human Papillomavirus (HPV) vaccine generates high antibody titers against targeted HPV types. This study investigated vaccine-induced anti-HPV18 immunoglobulin (IgG) antibody titers and subsequent HPV18/45 infections. **Methods:** We performed a nested matched case-control study leveraging a prospective longitudinal cohort of adolescent and young adult women (AYW) vaccinated with the quadrivalent HPV vaccine (4vHPV) attending the Mount Sinai Adolescent Health Center (MSAHC) in Manhattan, NY. The case individuals included AYW who had an incident detection of cervical HPV18 (*n* = 3) or HPV45 (*n* = 34) DNA after vaccination and were compared to two vaccinated control individuals (HPV18/45-negative); one random control (RC, *n* = 37) and one high-risk control (HRC, *n* = 37) selected from the upper quartile of a sexual risk behavior score. Serological titers against HPV18 were measured by end-point dilution and enzyme-linked immunosorbent assay (ELISA) in serum collected before the incident detection of HPV. Matching was performed based on age at first dose, follow-up time, and sexual risk behavior score. Conditional logistic regression was used to assess the association between case-control status and anti-HPV antibody titers, consistent with the matched-pair design. **Results:** Antibody titers for HPV18 were most different between AYW who developed an HPV18/45 infection compared to high-risk controls OR = 1.66, 95% CI: 0.96–2.85 (*p* = 0.1629). Analyses of pooled data from vaccinated recipients including who developed HPV16/31 or HPV18/45 infections demonstrated that the odds of a one-log unit increase in anti-HPV16 or 18 antibody titers, respectively, were 40% higher in the combined control groups (RC + HRC, *n* = 160) (OR = 1.40, 95% CI: 1.09–1.79, *p* = 0.0135) and 73% higher in the HRC (*n* = 80) (OR 1.73, 95% CI: 1.34, 2.52, *p* = 0.0117) compared to HPV16/18/31/45 cases (*n* = 80). **Conclusions**: Overall, these findings suggest that higher IgG antibodies to HPV16/18 after vaccination represent an increased likelihood of protection from homologous and cross-reactive HPV types (HPV16/18/31/45). These results show that differences in antibody titers are associated with breakthrough infection after vaccination, suggesting that further study of long-term antibody titers and infection should be pursued.

## 1. Introduction

Cervical cancer is the fourth most common cancer among women globally, with an estimated number of 660,000 new cases and 350,000 deaths in 2022 [1]. Nearly all cervical cancers are caused by persistent infections with high-risk human papillomavirus (HPV) genotypes, including HPV 16, 18, 31, 33, 35, 39, 45, 51, 52, 56, 58, 59, and 68. The introduction of prophylactic vaccines targeting the two most oncogenic HPV types, HPV16 and HPV18, demonstrated that cervical cancer is largely preventable [2,3]. The prophylactic HPV vaccines are composed of non-infectious virus-like particles (VLPs) formed from type-specific recombinant L1 major capsid proteins. These VLPs elicit a robust immune response, characterized by the production of neutralizing antibodies (NAbs). Upon subsequent HPV exposure, type-specific NAbs bind to the virus and block its entry into host target cells, preventing infection and disease. Additionally, the L1 proteins contain shared epitopes across phylogenetically similar HPV types, enabling the production of cross-reactive NAbs that confer partial protection against related non-vaccine HPV types [4,5].

The quadrivalent HPV vaccine (4vHPV, Gardasil) has demonstrated high efficacy in preventing persistent infections and cervical lesions caused by vaccine-targeted types, supported by clinical trials and observational studies [6,7,8,9]. Nearly all vaccinated individuals seroconvert, generating high titers of vaccine-type antibodies, with some cross-reactive antibodies to phylogenetically related non-vaccine types, albeit at lower levels. These vaccine-induced antibody titers are significantly higher and more durable than those elicited by natural infection and have been shown to persist for at least a decade [10,11]. Despite this robust immune response, breakthrough infections with vaccine HPV types have been documented [12,13]. The mechanisms underlying these breakthrough infections remain poorly defined [5], and there is currently no established serological threshold that reliably predicts protection. [10] Moreover, emerging evidence suggests immune functions beyond neutralizing antibodies might contribute to the vaccine’s high efficacy [14,15]. Real-world evidence is urgently needed, especially amongst sexually active individuals, to better understand the importance of antibody titers and inform vaccine recommendations.

We previously conducted a nested case-control study evaluating anti-HPV16 antibody titers prior to incident HPV16/31 infection in a diverse cohort of vaccinated adolescent and young adult women (AYW), finding that higher antibody titers were associated with enhanced protection [16]. In the present study, we expand this work by investigating whether anti-HPV18 antibody titers similarly correlate with protection from infections with HPV18 or HPV45 (phylogenetically related to HPV18) in vaccinated AYW. We hypothesized that higher anti-HPV18 antibody titers would be detectable among vaccinated AYW “controls” (i.e., uninfected) compared to “cases” (i.e., AYW who acquired an incident infection after vaccination). We then pooled our data from both studies to assess the role of anti-HPV16/18 antibody titers after vaccination in predicting protection against subsequent infection with HPV16/18/31/45.

This study examines the association between post-vaccination antibody titers and incident HPV infection in a real-world, longitudinal setting of sexually active young women. By focusing on antibody levels prior to breakthrough infection, we aim to better understand the potential role of serum IgG antibody titers as a biomarker of protection and cross-protection, particularly in high-risk populations where vaccine efficacy may be attenuated.

## 2. Materials and Methods

### 2.1. Study Population

Study participants were selected from an ongoing longitudinal HPV cohort study of AYW taking place at the Mount Sinai Adolescent Health Center (MSAHC) in New York City, as previously described [17,18]. The study was initiated in 2007 following the approval of the first HPV vaccine. While data collection for the overall cohort is ongoing, this nested case-control analysis includes individuals enrolled between 2007 and May 2021.

The study participants included sexually active AYW aged 13–21 years at the time of enrollment who intended to receive or had already received the quadrivalent HPV vaccine (4vHPV; GARDASIL^®^, Merck & Co., Inc., Kenilworth, NJ, USA) targeting HPV types 6, 11, 16, and 18. Study participants received a gynecological exam at each visit and completed a self-reported questionnaire on the participants’ sexual behaviors, history of sexually transmitted infections (STIs), pregnancy, and contraceptive use. Specimen collection was performed at each 6-month visit by MSAHC healthcare providers until participants reached 26 years of age. Serum samples were collected either during the enrollment visit or within six months of receiving the third dose of the vaccine, followed by recurring serum collections every two years for all returning participants. Additional study design and protocol details are described elsewhere [18,19,20]. Written informed consent was obtained from all study participants prior to enrollment. The study was approved by the Institutional Review Board at the Icahn School of Medicine at Mount Sinai, Manhattan, New York.

### 2.2. Cases and Controls

We performed a nested matched case-control study. The criteria for the case group were an incident detection of cervical HPV18 (*n* = 3) or HPV45 (*n* = 34) after three doses of 4vHPV amongst baseline HPV18/45 DNA-negative individuals. Two vaccinated control individuals per case were selected: one random control (RC) and one high-risk control (HRC). The RC was defined as an individual who was HPV18/45-negative at the time of incident detection, irrespective of their dose level. The RC was matched to the case based on age at first dose of 4vHPV (± one year) and follow-up time after receiving the last dose, irrespective of their sexual risk behavior score (SBS) level. The HRC was defined as a participant who received three doses of 4vHPV, was HPV18/45-negative at the time of the case’s incident detection, and was in the upper quartile of a sexual risk behavior score (score > 9) [16]. The sexual risk behavior score was derived based on variables (lifetime vaginal sex partners, number of recent (past six months) partners, history of Chlamydia trachomatis infection, any history of pregnancy, emergency contraception use, ever had anal sex, and lifetime number of anal sex partners) shown in Table 1. The HRC was matched to the case based on age at the first dose of 4vHPV (± one year) and follow-up time after receiving the third dose. Additional details on study variables have been described elsewhere [16].

### 2.3. HPV Testing

The incident detection of cervical HPV18 and HPV45 was performed as part of the longitudinal cohort study, using a previously described protocol based on the MY09/MY11 polymerase chain reaction (PCR) detecting over 40 HPV types common to the cervicovaginal region. Testing was performed on cervical Pap specimens collected at baseline (enrollment) and each follow-up clinic visit. Specifics on specimen collection, HPV DNA detection, and typing have been previously described [18].

### 2.4. IgG-Specific Anti-HPV18 L1 VLP-Based Enzyme-Linked Immunosorbent Assay (ELISA)

The anti-HPV18 L1 VLP particles were generated according to published protocols [21,22] at the HPV Immunology Laboratory, Frederick National Laboratory for Cancer Research, Frederick, MD, USA. Total levels of anti-HPV18 IgG antibodies (both neutralizing and non-neutralizing) were measured using an enzyme-linked immunosorbent assay (ELISA) as previously described [21,23] with slight modifications. The PolySorp Nunc-Immuno plates (Thermo Fisher Scientific, Waltham, MA, USA) were coated with 2.7 mg/mL of HPV18 VLPs (100 μL/well) and incubated overnight at 4 °C. Then, the 96-well plates were washed with a 1x phosphate-buffered saline (PBS, Sigma, St. Louis, MO, USA) solution with 0.05% Tween 20 (Thermo Fisher Scientific, Waltham, MA, USA), followed by a 3 h incubation at room temperature with blocking buffer containing 4% non-fat dry milk (BioRad, Hercules, CA, USA) and 0.2% Tween 20 (Thermo Fisher Scientific, Waltham, MA, USA) in 1x PBS pH 7.4 (Sigma, St. Louis, MO, USA). The plates were washed again, and incubated with the serially diluted serum samples for 2 h at 37 °C. Serum samples were tested in sample buffer at a starting dilution of 1:200, and then serially diluted in 2-fold increments until 1:102,400 (100 μL/well); all dilutions were tested in duplicate. After subsequent washes, a 1:10,000 solution of goat anti-human IgG (Fcγ fragment specific) conjugated with HRP (Jackson Immunoresearch Laboratories, Inc., West Grove, PA, USA) in sample buffer was added for one hour at 37 °C. The plates were then developed with ABTS peroxidase substrate (Kirkegaard & Perry Laboratories, Inc., Gaithersburg, MD, USA) for 25 min at room temperature, and the reaction was stopped by adding 1% SDS (Kirkegaard & Perry Laboratories, Inc., Gaitherburg, MD, USA). The absorbance was measured at 405 nm with a reference wavelength of 490 nm in an automated microtiter plate reader (Molecular Devices, Menlo Park, CA, USA). Anti-HPV18 antibody titers were calculated from serum titration curves, taking the inverse of the interpolated serum dilution analogous to the ELISA negative cut-off value, defined as 0.2 OD units as previously described [24]. A larger serum titer value (including log-transformed) corresponds to a greater anti-HPV antibody titer, meaning antibodies to HPV18 VLPs were detected at a higher sample dilution. Each plate tested included a ‘participant standard’, one positive, and one negative control (all provided by the HPV Immunology Laboratory, Frederick National Laboratory for Cancer Research, Frederick, MD, USA), and a ‘no sample’ control.

To minimize assay variability, we utilized the standardized ELISA protocol from the HPV Serology Lab. The HPV18 VLPs and reference reagents used in our study were also from the HPV Serology Lab, which is currently leading international efforts in serological assay standardization [10].

### 2.5. Serum Samples

Available serum samples from the cases were tested at three time points: (1) immediately before the visit detecting incident cervical HPV18/45 (pre-infection, *n* = 37, average (standard deviation, SD) time since the last dose 3.6 (2.5) years); (2) at the time of incident detection of cervical HPV18/45, *n* = 37, average time since last dose 4.8 (2.7) years (only 11 serum samples available); and (3) after cervical HPV18/45 incident detection (post-infection, *n* = 25, average time since the last dose 6.0 (2.6) years). Serum samples from controls were only tested at one time point, matched by time-to-case pre-infection samples (*n* = 74, average time since last dose of 4vHPV of random and high-risk controls was 4.4 (2.5) years and 4.5 (3.5) years, respectively).

### 2.6. Statistical Analysis

Participant characteristics were numerically summarized using descriptive statistics. Continuous variables were described using mean (standard deviation, SD), and non-normal variables were log-transformed. The difference in continuous variables (‘Age at Study Enrollment’, ‘Age at Coitarche’, ‘Age at First 4vHPV Dose’, ‘Time since last 4vHPV Dose to pre-infection visit’, ‘Log Anti-HPV18 Antibody Titer at pre-infection visit’, ‘Log Anti-HPV18 Antibody Titer at pre-infection visit Case vs. Random Control’, ‘Log Anti-HPV18 Antibody Titer at pre-infection visit Case vs. High-Risk Control’) between the cases and controls was evaluated using analysis of variance (ANOVA) and t-tests. Nonparametric Kruskal–Wallis and Wilcoxon rank-sum tests were used for those data that violated normality even after transformation (‘Follow-up Time’, ‘Anti-HPV18 Antibody Titer at pre-infection visit’). Categorical participant characteristics (‘Race’, ‘Lifetime Number of Partners’, ‘Number of Past Partners’, Chlamydia Trachomatis’, ‘Any Pregnancy’, ‘Emergency Contraception’, ‘Anal Sex ’, and ‘Lifetime Number of Anal Sex Partners’, ‘First Vaccine Dose before Coitarche’) were represented as frequency counts and percentages, and their associations were examined using the Cochran-Mantel-Haenszel modified ridit score test.

The association between exposure log anti-HPV antibody titer values and case-control status (HPV18/45, HPV45, HPV16/18, HPV31/45, and HPV16/18/31/45) was investigated using a multivariable conditional logistic regression model adjusted for whether participants were vaccinated before coitarche (i.e., if age at first dose was before the age at first vaginal intercourse). We applied the Benjamini-Hochberg procedure to adjust for multiple testing using the false discovery rate (FDR) method.

A piecewise linear mixed-effects model analysis with random coefficients (intercept and slope) was fitted to study the trajectory and determine the changes in log anti-HPV antibody titer before and after infection among the cases. First, actual time was calculated between the last vaccine dose date and the pre-, peri-, and post-infection samples. Then, (relative) time in the model was adjusted to reflect the duration since infection, which could result in positive and negative values. The estimation used a restricted maximum likelihood approach with Kenward–Roger degrees of freedom adjustment. The linear mixed-effects models allow for the fit of inherently unbalanced repeated measures data and missing information, assuming the data were “missing at random.” SAS software version 9.4 (SAS Institute, Cary, NC, USA) was used for all analyses. All data were analyzed in 2024–2025.

## 3. Results

A total of 45 (3.4%; 95% CI: 2.5–4.6) incident HPV18 or HPV45 infections were detected amongst 1306 AYW in the MSAHC HPV study cohort from study enrollment to May 2021. Of these 45, 37 (2.8%; 95% CI: 2.0–3.9) participants developed the infection subsequent to receiving all three doses of 4vHPV. These 37 participants were selected as the cases for this nested case-control study and were compared with two controls: (1) a random control group (RC) and (2) a high-risk control group (HRC). The distribution of participant characteristics, including the cases (*n* = 37) and their matched controls (*n* = 74) is presented in Table 1.

No statistical difference was observed in age at study enrollment, age at receipt of first 4vHPV dose, and race of participants between the case and control groups. The participants were 17.8± 1.5 (SD) years old at study enrollment and received their first 4vHPV vaccine at 14.7 ± 2.2 (SD) years. The majority (107 out of 111) identified as Hispanic or African American. As expected, the cases and controls were significantly different in their sexual risk behavior scores and associated variables, such as number of lifetime sexual partners, lifetime anal sexual partners, past sexual partners, and frequency of contraceptive use. The mean age at coitarche was 14.6 ± 1.2 (SD) for the cases, 15.1 ± 1.5 (SD), and 14.0 ± 1.8 (SD) for the RCs and HRCs, respectively, and about half of the cases and RCs (51% and 46%, respectively) initiated sexual activity after they had received at least one dose of the vaccine compared with only 19% of the HRCs.

### 3.1. Comparison Between Cases and Controls at the Pre-Infection Visit

**Figure 1:**
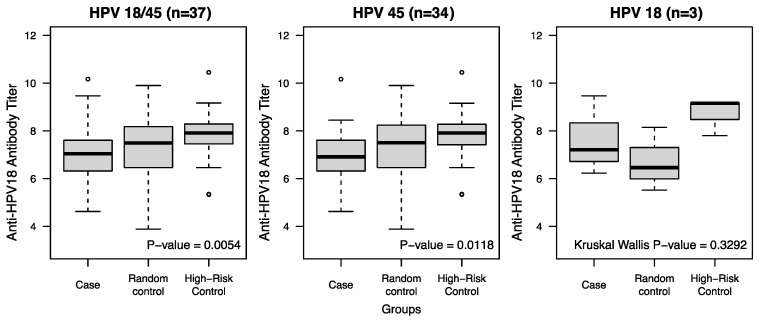
Distribution of log anti-HPV18 antibody titers according to case-control group at pre-infection visit. Boxplots illustrate the distribution of log anti-HPV18 antibody titers measured at pre-infection visit (or corresponding visit for controls) by group as defined below each panel. (**Left**), HPV18 and HPV45 incident cases with matching controls (each group *n* = 37); (**Middle**), HPV45 incident cases only with matching controls (each group *n* = 34); (**Right**), HPV18 incident cases only with matching controls (each group *n* = 3). Cases and controls were tested using ANOVA except for the HPV18-only panel, which was tested using the Kruskal–Wallis test. The *p*-value is shown at the bottom right corner of each panel.

The median anti-HPV18 antibody titers and the mean log anti-HPV18 antibody titers for each group are displayed in Table 1. The average log anti-HPV18 antibody titer values were 7.0, 7.2, and 7.9 for the cases, RCs, and HRCs, respectively. The difference across the groups was significant at the *p* = 0.005 level using the analysis of variance. When comparing the mean log anti-HPV18 antibody titer of cases to each control group separately, they were significantly different only to the HRC (*p* = 0.0007), but not to the RC (*p* = 0.2487) (Table 1). Separated by HPV type, the difference between the log-transformed anti-HPV18 antibody titers across cases and controls was significant only for HPV45 (*n* = 34), and not for the HPV18 cases (*n* = 3), but the low sample size of HPV18 cases might have driven this difference (Figure 1). The FDR-adjusted *p*-values for differences in anti-HPV titers between cases and controls were 0.0162 for HPV18/45, 0.0177 for HPV45, and 0.3292 for HPV18, respectively.

To assess the association between anti-HPV18 antibody titers after vaccination and case-control status (reflecting subsequent HPV18/45 infection), a conditional logistic regression analysis was performed, adjusted for having initiated sexual activity after taking the 4vHPV vaccine. The results showed that the controls (RC + HRC) had higher antibody titers, having 37% higher odds of a one-log unit increase in anti-HPV18 antibody titers compared to the cases (OR = 1.37, 95% CI: 0.93–2.02; *p* = 0.1629), as shown in Table 2. Similar results were observed when the associations were analyzed separately for each control group. The association between anti-HPV18 antibody titers and case-control status was highest for the HRC, which showed 66% higher odds of a one-log unit increase in antibody titers compared to the cases (OR = 1.66, 95% CI: 0.96–2.85; *p* = 0.1629). Thus, though not statistically significant, AYW with high sexual risk behaviors who did not acquire HPV 18/45 infections had higher titers, implying these titers protected them from infection.

In a subgroup analysis for only HPV45 case-control pairs, the effect did not reach statistical significance for HPV45 Cases and Controls (RC + HRC), where controls were 52% more likely than cases to have a one-log unit increase in anti-HPV18 antibody titers (OR = 1.52, 95% CI: 0.98–2.38; *p* = 0.1362). Overall, similar trends were observed in the HPV18/45 and subgroup (HPV45) analyses: RC and HRC had higher odds of a one-log unit increase in anti-HPV18 antibody titers compared to the cases analyzed as a composite control group (RC + HRC), and individually (RC or HRC).

A pooled dataset of anti-HPV16 antibody titers prior to HPV16/31 infections from our previous publication in the same cohort [16] and anti-HPV18 antibody titers prior to HPV18/45 infections from the current study was used to estimate the association between case-control status and log anti-HPV16/18 antibody titers. HPV16/18/31/45 cases were analyzed as a composite and as subgroups HPV16/18 and HPV31/45. Adjusting for whether participants were vaccinated before coitarche, the odds of having a one-log unit increase in anti-HPV16/18 antibody titer were 40% higher among controls (RC + HRC) (*n* = 160) than cases (*n* = 80) for HPV16/18/31/45. When comparing cases (*n* = 80) only to the HRCs (*n* = 80), the odds were 73% higher for the HR controls. Among HPV31/45 cases and their controls, the relative odds were 59% higher among controls (*n* = 102), and 78% higher when comparing the HPV31/45 cases only to the HRCs (*n* = 51). There were no significant differences in antibody titers between cases and controls when limited to HPV16 and HPV18 (Table 3), although the numbers were small.

### 3.2. Subgroup Analysis: A Longitudinal Evaluation

To assess the influence of the incidence of HPV18/45 infection on the anti-HPV18 antibody titer, we compared the log anti-HPV18 antibody titers in the serum collected immediately before (pre-infection), at (peri-infection), and after (post-infection) the detection of HPV18/45. The pre-infection, peri-infection, and post-infection sera were collected on average 3.6, 4.8, and 6.0 years after receiving the last vaccine dose. On average, the pre-infection sera were collected 1.25 years prior to HPV detection, and the post-infection sera were collected 1.53 years after HPV detection. The average log anti-HPV18 antibody titers remained relatively stable over time in the three visits evaluated (Table 4).

Based on the piecewise linear mixed model, the average log anti-HPV18 antibody titers increased from pre- to peri-infection by 0.1 units (standard error, SE: 0.11), although not statistically significant (*p* = 0.38). The estimated increase in slope is relatively low, corresponding to an approximate 10% (i.e., e^0.095^ = 1.10) increase in serum titers on the actual scale. However, the estimated mean log serum titers showed a decrease of 0.045 (SE: 0.11) (∆β = 0.095–0.14) units from peri- to post-infection, a 5% (= e^−0.05^) decrease in serum titers on the actual scale, and the change was non-significant (*p* = 0.68). The results from the regression model are shown in Table 5. This case analysis suggests that potential “false negative” undetected HPV infections post-vaccination were not driving the larger antibody titers detected in the HRC group.

## 4. Discussion

Following our previously published study [16], we expanded our investigation to explore if the anti-HPV18 antibody titers were lower in serum samples from immunized AYW before an incident cervical HPV18/45 infection compared to matched individuals without detectable HPV18/45 infection. HPV18 cases were included as a direct vaccine-targeted type, while HPV45 cases were included as a closely related non-vaccine type. Due to their phylogenetic similarity and structural homology [25,26], and evidence that antibodies elicited by the 4vHPV vaccine can neutralize HPV45 in vitro [27], HPV45 may be subject to cross-protection via anti-HPV18 antibodies. Thus, grouping HPV18 and 45 allowed us to assess whether antibody levels conferred protection not only against the vaccine-targeted type, but also against a related non-vaccine type.

Our results indicated that the IgG anti-HPV18 antibody titers were lower among incident HPV18/45 cases compared to controls. However, the association between anti-HPV18 antibody titer and case-control status for HPV18/45 did not reach statistical significance in this study, likely due to the low sample size. Pooling our data of case-control pairs for all four high-risk types (HPV16/18/31/45), we show that post-vaccination anti-HPV16/18 antibody titers were significantly higher among the controls than matched cases prior to breakthrough infections with HPV16/18/31/45. Thus, higher anti-HPV16/18 antibody titers after vaccination may be associated with a reduced risk of breakthrough infections with homologous vaccine types and cross-related HPV types not targeted by 4vHPV. While we cannot definitively conclude that the anti-HPV16/18 Abs are directly responsible for the observed protective effect, our findings suggest that their titers contribute to it, at least in part. This is biologically plausible as IgG Abs to HPV VLPs measured by ELISA serve as a reliable surrogate for NAbs [28], the primary effectors of vaccine-induced protection. Furthermore, these titers likely represent both type-specific neutralization (in the case of HPV16 or HPV18) and cross-neutralization (in the case of HPV31 or HPV45).

The 4vHPV vaccine has shown partial cross-protective efficacy against phylogenetically related non-vaccine types HPV31 and HPV45 [29]. In subgroup analyses restricted to non-vaccine case-control pairs, we found significantly higher anti-HPV16/18 antibody titers in controls than in cases prior to infection with HPV31/45 (OR = 1.59 (95% CI: 1.12–2.24) *p* = 0.0206). Our findings suggest that the detected amount of anti-HPV16/18 antibodies may at least be partially linked to the cross-protection levels against closely related types. Prior research showed that individuals vaccinated with HPV16/18 generated Abs to HPV31/45 that were correlated with levels of the related type [30]. Thus, the higher titers observed in our study likely represent higher cross-reactive antibodies to the related types, which are mediating cross-protection. Furthermore, cross-reactive Abs to HPV31 and HPV45 have been detected in vaccinated cohorts in the past, and have coincided with evidence of cross-protection at the population level of vaccinated individuals [4,29,31]. Our findings now provide a direct association between vaccine-type antibody titers after 4vHPV vaccination and protection from subsequent infection with cross-related types within the same individuals. Moreover, our results are consistent with prior research from the Costa Rica Vaccine Trial (CVT), which found lower anti-HPV16 antibody titers prior to infections with HPV31 among participants vaccinated with the bivalent HPV vaccine (2vHPV, Cervarix), compared to vaccinated HPV-negative controls [32]. The results reported here further expand on the relationship between IgG antibody titers and breakthrough infections by showing a quantitative association between titers and case-control status in a diverse population of sexually active AYW outside of the clinical trial setting. Here we investigate the relationship between antibody titer and subsequent infection among participants vaccinated with the 4vHPV, which has been shown to elicit differential immune responses compared to the 2vHPV, especially in terms of cross-protection [29,30,33].

Interestingly, the subgroup analysis for vaccine types HPV16/18 revealed a reduced and non-significant association between case-control status and antibody titer (OR = 1.18, 95% CI: 0.81–1.72, *p* = 0.5673) compared to protection for HPV31/45. Although this could be attributed to sample size limitations, we hypothesize that the significant association observed for the HPV31/45 case-control pairs represents the importance of titers for cross-reaction and protection, whereas for the cognate types, a broad range of titers is protective. In fact, one report found that cross-protective Abs to HPV31, HPV33, and HPV45 were <1% of the corresponding HPV16 and HPV18 antibody levels induced by the 2vHPV vaccine [34]. Therefore, a reduction in antibody levels to HPV16/18 corresponds to an even greater reduction in cross-protective antibodies (HPV31/45), possibly falling below the threshold of protection, which is conceivably below the detection limits of current serological assays [35]. Hence, participants with lower anti-HPV16/18 antibodies might be more susceptible to infection with HPV31/45, as shown in this study. Conversely, the 4vHPV induces a much higher and sustained antibody response against HPV16/18 [30], which may make it more difficult to observe an association between antibody titers and breakthrough infections with HPV16/18. For instance, in a longitudinal cohort of Dutch women, there was no consistent difference in type-specific antibody titers between cases one year before breakthrough infections and HPV-negative controls for both vaccine and non-vaccine types [36]. Instead of directly measuring type-specific antibody titers against HPV31/45, our study assessed anti-HPV16/18 antibody titers, which might act as proxies for the lower level of type-specific antibodies. Alternatively, anti-HPV16/18 antibody titers could be markers for other associated immunological responses, such as cell-mediated B-cell or T-cell responses influencing cross-protection. This might explain why we could detect differences between HPV31/45 cases and HPV-negative controls, whereas such differences were not observed in the Dutch cohort. Moreover, our study differed in vaccine type and study design, where we matched cases with controls on age at the first vaccine dose and follow-up time and adjusted the logistic regression models according to sexual activity before vaccination.

High-risk sexual behavior is considered a main risk factor for the acquisition and persistence of HPV infection and the development of HPV-related cancers [37,38,39]. The strongest associations between antibody titer and case-control status in all the analyses were observed when high-risk controls (HRC) were compared to cases. That is, HRC had 73% higher antibody titers than HPV16/18/31/45 cases (95% CI: 1.34–2.52), while the antibody titers for both control groups combined (RC + HRC) were only 40% higher than HPV16/18/31/45 cases (95% CI: 1.09, 1.79). These results show that the absence of breakthrough infection in the HRC group, despite their innate higher risk, corresponds with even higher antibody titers against HPV16/18/31/45 compared to those in the randomly selected controls. While we cannot exclude the possibility that the higher titers are induced by frequent HPV exposure within this group, the longitudinal analyses of the HPV18/45 cases, which showed no boost in antibody levels post-infection, suggest that the high titers may not be due to undetected HPV breakthrough infection after vaccination. Therefore, with the similarity in trends observed for HRC and for non-vaccine types (HPV31/45), we infer that antibody IgG titers may serve as quantitative markers of protection, specifically in scenarios where participants may already be more susceptible to breakthrough infections via sexual behaviors or among groups where vaccine efficacy is attenuated, i.e., immunocompromised, autoimmune disorders, or solid organ transplant patients [40].

Overall, the associations identified in this study indicate that monitoring anti-HPV16/18 antibody titers in vaccinated cohorts, as possible biomarkers for immunity, could help estimate protection levels against both vaccine-type and related non-vaccine-type HPV, especially in high-risk individuals, and could inform booster vaccination schedules. While there still is no established protective threshold for HPV antibody titers, antibody titers are already used in immunobridging studies and are becoming increasingly important as new vaccines are developed [41]. Recently, anti-HPV antibody titers following vaccination to treat HPV genital infection in men were correlated to HPV clearance and proposed as prognostic markers of HPV resolution [42]. More research with a larger sample size is needed on vaccine failures to establish a serological protection threshold and derive clinical implications from antibody titers after vaccination. We provide a critical foundation for these efforts with valuable real-world evidence from a sexually active cohort of vaccinated women outside of a clinical trial setting. Our findings support the high immunogenicity of the 4vHPV and its cross-protective effects against HPV31/45, mediated by cross-reactive antibodies assessed by anti-HPV16/18 antibody titers in our study.

One of the limitations of the present study is the limited sample size. The prevalence of cervical HPV types either targeted by the 4vHPV vaccine (HPV6/11/16/18), or by cross-protection (HPV31/45) dropped from 9.1% between 2008 and 2010 to 4.7% between 2017 and 2019 (*p* = 0.004) in the MSAHC cohort [17] from which the cases and controls of this study were selected; hence, the number of cases available for our nested case-control sub-studies was limited. Moreover, the patient population of the MSAHC is heavily composed of self-identified Hispanics and African Americans. Therefore, our longitudinal cohort and the selection of participants for our case-control study reflect the demographics of the clinic, limiting the generalizability of our results to other ethnic groups or the broader U.S. population. Evidence on how efficacious HPV vaccines are in reducing cervical precancer burden is mounting [3,43,44]. However, this does not diminish the significance of our findings, which become increasingly crucial in predicting rare breakthrough HPV cases. Lastly, being sexually active was considered a criterion for inclusion in the prospective large cohort study, which means that there was likely HPV exposure before enrollment and possibly at the time of vaccination.

## 5. Conclusions

This study demonstrates that among vaccinated AYW, lower anti-HPV16/18 antibody titers pose a higher risk for subsequent HPV16/18/31/45 infections. Our findings highlight the importance of monitoring antibody titers as a correlate of vaccine-induced protection over time.

## Figures and Tables

**Table 1:** Descriptive Statistics of Case and Control Groups.

Variables	Cases (*n* = 37)	Random Controls (*n* = 37)	High-Risk Controls (*n* = 37)	*p*-Value
Age at Study Enrollment Mean (years ± SD)	17.76 ± 1.56	17.94 ± 1.30	17.79 ± 1.54	0.8550
Age at Coitarche Mean (years ± SD)	14.57 ± 1.21	15.05 ± 1.51	13.97 ± 1.83	0.0124
Age at First 4vHPV Dose Mean (years ± SD)	14.18 ± 2.23	14.70 ± 2.23	14.65 ± 2.10	0.9648
Race *n* (%)				
Hispanic	19 (51.35)	22 (59.46)	21 (56.76)	0.6264
African American	16 (43.24)	13 (35.14)	16 (43.24)	
Other	2 (5.41)	2 (5.41)	0 (0)	
Lifetime Number of Partners *n* (%)				
1	0 (0)	3 (8.11)	0 (0)	0.0027
2	4 (10.81)	7 (18.92)	0 (0)	
3	12 (32.43)	8 (21.62)	6 (16.22)	
4+	21 (56.76)	19 (51.35)	31 (83.78)	
Number of Past Partners (6 months) *n* (%)				
0	2 (5.41)	1 (2.70)	0 (0)	0.0254
1	23 (62.16)	23 (62.16)	15 (40.54)	
2+	12 (32.43)	13 (35.14)	22 (59.46)	
Chlamydia Trachomatis *n* (%)				
Yes	21 (56.76)	20 (54.05)	32 (86.49)	0.8073
Any Pregnancy *n* (%)				
Yes	17 (45.95)	12 (32.43)	17 (45.95)	0.3986
Emergency Contraception Ever *n* (%)				
Yes	30 (81.08)	26 (70.27)	37 (100)	0.0022
Anal Sex Ever *n* (%)				
Yes	18 (48.65)	15 (40.54)	35 (94.59)	<0.0001
Lifetime Number of Anal Sex Partners *n* (%)				
0	19 (51.35)	22 (59.46)	2 (5.41)	<0.0001
1	12 (32.43)	6 (16.22)	13 (35.14)	
2+	6 (16.22)	9 (24.32)	22 (59.46)	
Sexual Risk Behavior Score Mean ± SD	7.70 ± 2.31	7.11 ± 2.60	10.30 ± 1.10	<0.0001
First Vaccine Dose Before Coitarche *n* (%)				
Yes	19 (51.35)	17 (45.95)	7 (18.92)	0.0094
Follow-up Time (years)				
Median (Min-Max)	2.86 (0.60–8.28)	6.26 (1.71–10.00)	6.16 (0.84–8.87)	0.0017
Time since last 4vHPV Dose to pre-infection visit Mean (years ± SD)	3.6 ± 2.5	4.4 ± 2.5	4.5 ± 3.5	0.3111
Anti-HPV18 Antibody Titer at pre-infection visit, Median	1144.74	1793.35	2729.56	
(Min-Max)	(102.09–26,039.36)	(48.82–19,952.51)	(207.58–34,589.68)	<0.0001
Log Anti-HPV18 Antibody Titer at pre-infection visit Mean ± SD	7.01 ± 1.14	7.24 ± 1.38	7.88 ± 0.98	0.0054
Log Anti-HPV18 Antibody Titer at pre-infection visit Mean ± SD				
Case vs. Random Control	7.01 ± 1.14	7.24 ± 1.38	-	0.2487
Log Anti-HPV18 Antibody Titer at pre-infection visit Mean ± SD				
Case vs. High-Risk Control	7.01 ± 1.14	-	7.88 ± 0.98	0.0007

SD = standard deviation.

**Table 2:** Association between Log Anti-HPV18 Antibody Titers and Case-Control Status stratified by Case HPV Types.

	HPV18/45 (*n* = 37)			HPV45 Only (*n* = 34)		
	OR (95% CI) ^§^	*p*-Value	FDR *p*-Value	OR (95% CI) ^§^	*p*-Value	FDR *p*-Value
Controls (RC + HRC) vs. Cases	1.37 (0.93–2.02)	0.1086	0.1629	1.52 (0.98–2.38)	0.0647	0.1362
RC vs. Cases	1.18 (0.40–4.17)	0.4459	0.4454	1.33 (0.82–2.16)	0.2453	0.2453
HRC vs. Cases	1.66 (0.96–2.85)	0.0673	0.1629	1.60 (0.93–2.77)	0.0908	0.1362

^§^ Odds ratio (OR) (95% confidence intervals, CI) estimated by multivariate conditional logistic regression, adjusting for vaccination before coitarche; the fitted regression models probability (relative odds) of control having one-log unit increase in anti-HPV18 antibody titers compared to the cases; HPV, human papillomavirus; RC, random control; HRC, high-risk controls. The association between log serum titers and HPV18 incident cases was not assessed due to the small number of cases. FDR *p*-value: Benjamini-Hochberg false discovery rate adjusted *p*-value.

**Table 3:** Association between Log Anti-HPV16/18 Antibody Titers and Case-Control Status stratified by Case HPV Types.

	HPV16/18/31/45 (*n* = 80)			HPV16/18 Only (*n* = 29)			HPV 31/45 Only (*n* = 51)		
	OR (95% CI) ^§^	*p*-Value	FDR *p*-Value	OR (95% CI) ^§^	*p*-Value	FDR *p*-Value	OR (95% CI) ^§^	*p*-Value	FDR *p*-Value
Controls (RC + HRC) vs. Cases	1.40 (1.09, 1.79)	**0.0090**	**0.0135**	1.18 (0.81, 1.72)	0.3782	0.5673	1.59 (1.12, 2.24)	**0.0089**	**0.0206**
RC vs. Cases	1.22 (0.93, 1.60)	0.1468	0.1468	1.02 (0.67, 1.54)	0.9348	0.9348	1.39 (0.96, 2.01)	0.0838	0.0838
HRC vs. Cases	1.73 (1.34, 2.52)	**0.0039**	**0.0117**	1.71 (0.87, 3.38)	0.1205	0.3615	1.78 (1.13, 2.80)	**0.0137**	**0.0206**

^§^ Odds ratio (OR) (95% confidence intervals, CI) estimated by multivariate conditional logistic regression, adjusting for vaccination before coitarche; the fitted regression models probability (relative odds) of a control having one-log unit increase in anti-HPV16/18 antibody titers compared to the cases; HPV, human papillomavirus; RC, random control; HRC, high-risk controls; FDR *p*-value: Benjamini-Hochberg false discovery rate adjusted *p*-value. Statistically significant *p*-values are bolded.

**Table 4:** Descriptive Statistics for Cases pre-, peri-, and post-infection.

Variables	Pre-Infection (*n* = 37) Mean ± SD	Peri-Infection (*n* = 37) * Mean ± SD	Post-Infection (*n* = 25) Mean ± SD
Log Anti-HPV Antibody Titers	7.01 ± 1.14	7.00 ± 1.30	7.02 ± 1.15
Duration of Time from Last Dose (years)	3.57 ± 2.54	4.82 ± 2.66	6.00 ± 2.59
Serum Collection Time Relative to Infection (years)	−1.25 ± 0.78	0 ± 0	1.53 ± 0.67

*n* is the number of cervical samples and serum samples tested; * indicates there were 37 cervical samples but only 11 serum samples for the peri-infection time; SD = standard deviation.

**Table 5:** Estimated Regression Coefficients (fixed and random effects).

Variables	β (SE)	*p*-Value
Intercept (mean log anti-HPV18 antibody titers at pre-infection)	7.10 (0.20)	<0.0001
Time (change from pre- to peri-infection)	0.095 (0.11)	0.3796
Time+ (additional change peri- to post-infection)	−0.14 (0.18)	0.4450
Random Effects		
Variance (random intercept)	0.92 (0.27)	0.0003
Variance (residual)	0.38 (0.09)	<0.0001
SE = standard error		

## Data Availability

The data that support the findings of this study are available upon reasonable request from the corresponding author.

## Abbreviations

The following abbreviations are used in this manuscript:

AYW	Adolescent and Young Adult Women
Abs	Antibodies
2vHPV	Bivalent HPV Vaccine
ELISA	Enzyme-linked immunosorbent assay
HRC	High-Risk Control
HPV	Human Papillomavirus
IgG	Immunoglobin G
MSAHC	Mt. Sinai Adolescent Health Center
NAbs	Neutralizing antibodies
4vHPV	Quadrivalent HPV vaccine
RC	Random Control
SBS	Sexual Risk Behavior Score
VLPs	Virus-Like Particles
